# Social Reward, Punishment, and Prosociality in Paranoia

**DOI:** 10.1037/abn0000647

**Published:** 2020-12-03

**Authors:** Nichola Raihani, Daniel Martinez-Gatell, Vaughan Bell, Lucy Foulkes

**Affiliations:** 1 Department of Experimental Psychology, University College London; 2 Department of Clinical, Educational, and Health Psychology, University College London; 3 Department of Education, University of York

**Keywords:** cooperation, economic games, paranoia, prosocial, punishment

## Abstract

Paranoia is the exaggerated belief that harm will occur and is intended by others. Although commonly framed in terms of attributing malicious intent to others, recent work has explored how paranoia also affects social decision-making, using economic games. Previous work found that paranoia is associated with decreased cooperation and increased punishment in the Dictator Game (where cooperating and punishing involve paying a cost to respectively increase or decrease a partner’s income). These findings suggest that paranoia might be associated with variation in subjective reward from positive and/or negative social decision-making, a possibility we explore using a preregistered experiment with U.S.-based participants (*n* = 2,004). Paranoia was associated with increased self-reported enjoyment of negative social interactions and decreased self-reported enjoyment of prosocial interactions. More paranoid participants attributed stronger harmful intent to a partner. Harmful intent attributions and the enjoyment of negative social interactions positively predicted the tendency to pay to punish the partner. Cooperation was positively associated with the tendency to enjoy prosocial interactions and increased with participant age. There was no main effect of paranoia on tendency to cooperate in this setting. We discuss these findings in light of previous research.

## General Scientific Summary

This study shows that paranoia is associated with alterations to the differential self-reported enjoyment from engaging in different kinds of social interaction. Specifically, more paranoid individuals reported enjoying positive prosocial interactions less and enjoying negative social interactions more. These alterations can help to explain variation in social behavior in controlled experimental settings.

Paranoia involves being troubled by excessive concerns that harm will occur and that this harm is intended by others (Freeman & Garety, 2000). Although paranoia traditionally referred to delusional paranoia, a broader spectrum of paranoid concerns also occurs in a range of psychiatric syndromes and throughout the general population where it can encompass perceptions of social reference and hostility, suspicious thoughts and mistrust of others, and fears of persecution (Bebbington et al., 2013; Combs et al., 2006; Elahi et al., 2017; Freeman, 2016; Green et al., 2008). Paranoia shows full taxometric continuity across clinical and nonclinical populations (Ahmed et al., 2012; Edens et al., 2009; Elahi et al., 2017), supporting a dimensional approach to paranoia and suggesting that studying the cognitive and affective correlates of paranoid thinking in the general population will yield insights into more severe paranoia in clinical settings (Freeman et al., 2005; Raihani & Bell, 2019). Our aim is to determine whether paranoia correlates with altered social reward value, and whether this predicts social behavior in an experimental setting.

Although factors that maintain clinical paranoia, such as sleep difficulties and worry, have been relatively well-characterized (Freeman, 2016), the mechanisms of social decision-making in paranoia have received much less attention. Recently, work using game theory paradigms that involve experimentally controlled strategic social interactions have begun to inform the social cognition of paranoid social inference and decision-making (Barnby et al., 2020; Fett et al., 2012; Greenburgh et al., 2019; Gromann et al., 2014; Raihani & Bell, 2018, 2017; Saalfeld et al., 2018). In particular, the Dictator Game (Kahneman et al., 1986), where one player (the dictator) receives money and can choose how much to send to the partner (the receiver), has been used to explore how variation in paranoia affects the perception of others’ intentions (Greenburgh et al., 2019; Raihani & Bell, 2017; Saalfeld et al., 2018). The motives underpinning dictator decisions remain ambiguous, but participants reporting high levels of paranoid ideation show an increased tendency to attribute harmful intent, but not self-interest, to dictators in this game (Barnby et al., 2020; Raihani & Bell, 2017), which is modified by the perceived group characteristics of the partner (Greenburgh et al., 2019; Saalfeld et al., 2018).

In addition to social inference, game-theoretic paradigms can also be used to explore how social decision-making varies in paranoia and in psychosis. Several studies have reported reductions in cooperation (Hanssen et al., 2018; Raihani & Bell, 2018) and trust (Fett et al., 2012; Gromann et al., 2013; Lemmers-Jansen et al., 2019) in paranoia. This has been conceptualized as the result of altered social reward learning mediated by changes to mesolimbic dopamine function (e.g., Diaconescu et al., 2019; Gromann et al., 2013). However, a key distinction rarely tackled by these studies is between the reward associated with a partner’s response, compared with intrinsic reward value associated with one’s own decisions (Harbaugh et al., 2007; Moll et al., 2006). The reward processes associated with one's own decisions need not be related to how the partner is expected to behave nor how rewarding experiencing cooperation from a partner might be. The subjective reward associated with experiencing cooperation from a partner versus making a cooperative decision oneself are not mutually exclusive, although the latter remains relatively understudied in paranoia.

We previously showed that paranoia involves an increased attribution of harmful intent to others and an increased tendency to punish selfish partners (Raihani & Bell, 2018). Punishment decisions are known to depend not just on whether harm occurred but on the perception of whether the harm was intended (Cushman et al., 2009; Gerstenberg et al., 2011; Sarin et al., 2017; Schaechtele et al., 2011). In this previous work, increased punitive tendency in paranoia was partly (but not fully) mediated by the tendency to attribute harmful intent to others. In the same study, more paranoid thinking was also associated with reduced prosocial tendency: more paranoid participants sent less money to the partner when acting in the role of the dictator. Together, these findings suggest that punitive decisions in paranoia might be associated with variation in subjective reward value associated with positive and negative social decision-making rather than simply an exaggerated perception of threat from others—a hypothesis we test here. We asked whether paranoia was associated with variation in the self-reported tendency to find different kinds of social interaction more or less enjoyable. We then asked to what extent paranoia, harmful intent attribution, and social reward value are associated with punishment and generosity (respectively) in an experimental Dictator Game.

## Method

### Participants

Data for this study were collected in April and May 2018. This project was approved by the UCL Research Ethics Council (project 3720/001). We recruited 2,004 U.S.-based participants (1,112 females, 892 males) using the online platform Amazon Mechanical Turk (hereafter, MTurk). The mean age of participants was 35.8 ± 0.3 (range: 18–82). All analyses in this study are preregistered, unless reported otherwise. All predictions for this study, as well as data and code to reproduce analyses, are available online at https://osf.io/6kv3d/.

### Procedure

#### Self-Reported Paranoia and Social Reward

We assessed participants for paranoia, using the Green et al. Paranoid Thought Scales (hereafter GPTS; Green et al., 2008), which is a reliable and valid instrument that captures variation in paranoid thinking across the full spectrum. The scale is described in online supplemental materials.

Participants then took two subscales of the Social Reward Questionnaire (SRQ), designed to measure individual differences in the hedonic value of different types of social interactions (Foulkes et al., 2014). All subscales of the SRQ are reliable, valid, and internally consistent and associate with related external factors (Foulkes et al., 2014). In this study, participants took the Negative Social Potency scale and the Prosocial Interactions subscales (hereafter NSP and PRO, respectively; described in the online supplemental materials), which each consisted of five items where participants rate on a Likert scale (1–7) the extent to which they agree or disagree with the statement. Higher scores indicate stronger self-reported enjoyment of antisocial and prosocial behavior, respectively. We expected that paranoia would be negatively associated with PRO and positively associated with NSP. All scales showed good internal consistency (Cronbach’s alpha: GPTS = 0.97; NSP = 0.92; PRO = 0.93). Participants were paid $0.70 for this part of the study.

#### The Experimental Task

After a minimum interval of seven days, participants were recalled and invited to take part in a modified Dictator Game (described below). Participants were allocated either to the role of receiver or dictator (within-subject, order counterbalanced). After a minimum interval of 10 days, participants were recalled again and assigned to the opposite role. Of the original 2,004 participants, we successfully rerecruited and obtained full responses from 1138/2004 participants in the dictator role and 1136/2004 participants in the receiver role.

Our first aim was to replicate previous findings that paranoia predicts increased harmful intent attribution but does not predict attributions of self-interest. We have successfully replicated these core findings several times before (Barnby et al., 2020; Greenburgh et al., 2019; Raihani & Bell, 2018, 2017; Saalfeld et al., 2018), allowing these analyses to act as a validity check for this study. We did successfully replicate these key findings here and present these in the supplementary information rather than in the main text.

#### Measuring Punitive Tendency

Our Dictator Game was modified to allow receivers to pay to punish their partners after seeing how much the partner decided to send, thereby allowing us to measure punitive tendency. We measured punitive tendency using the strategy method (Brandts & Charness, 2011; Fischbacher et al., 2012). Participants played in the receiver role in a Dictator Game, where they started with $0.05 and the dictator with $0.55. Participants were asked to state for every possible donation by the dictator, whether they would choose to punish or not. Punishment involved spending $0.05 to levy a fine of $0.15 on the dictator. Endowing receivers with $0.05 at the start of the task therefore meant that receivers could still afford to punish, even when dictators sent nothing to them. Receivers were truthfully informed that they would be matched with a real partner and that their punishment decision would be executed based on what the partner donated.

#### Measuring Generosity

Participants also played in the dictator role and could choose to send any amount to the partner (from $0.00 to $0.55 in $0.05 increments). In this game, participants knew that the receiver had a punishment option, as described above. We aimed to replicate our previous finding that paranoia would be associated with lower generosity in the Dictator Game (Raihani & Bell, 2018), as well as exploring whether NSP or PRO also explained variance in the decision to send money to the partner.

### Statistical Analyses

All data were analyzed using R package 3.5.0. Models were constructed as cumulative link models (clm) using the using the R package ordinal (Christensen, 2018). Commonality analyses were performed using R package yhat (Nimon et al., 2020). All statistical details available in the online supplemental materials. All deviations from planned analyses are reported in the online supplemental materials.

### Preregistered Predictions

1 Paranoia Is Positively Associated With Reward Value of Antisocial Behavior (NSP) and Negatively Associated With Reward Value of Prosocial Behavior (PRO)

We calculated the mean NSP and PRO scores for each participant and then converted these scores to ordinal categorical variables, each with 7 levels. These ordinal categorical variables were set as the response terms in two separate cumulative link models (clm), and we included paranoia, age, and gender as explanatory terms in each model.

2 Paranoia, Harmful Intent Attribution, and NSP Are Associated With Increased Punitive Tendency

We measured punitive tendency in two main ways: (a) punishment threshold (the maximum offer in the Dictator Game that participants said they would be willing to punish) and (b) total number of offers punished. These two variables were set as the respective ordinal categorical response terms (nine and eight levels, respectively) in two clms. For both models, we included the following explanatory terms: paranoia, NSP, harmful intent attribution, age, comprehension, gender, and order. We performed a commonality analysis to establish the unique and shared variance in the outcome variable explained by paranoia, harmful intent attribution, and NSP.

3 Paranoia, NSP, and PRO Are Associated With Decreased Generosity in the Dictator Game

Dictator donation was set as a six-level ordinal categorical response variable in a clm, with the explanatory terms paranoia, NSP, PRO, age, comprehension, and gender. We subsequently conducted a commonality analysis to explore the unique and shared explanatory value of paranoia, PRO, and NSP on the outcome variable.

## Results

Participants in this study spanned the full range for paranoid thinking captured by the GPTS (range: 32–160; mean: 55.3 ± 0.6). One hundred sixty-eight of the 2,004 (∼ 8%) participants scored higher than 101.9, the mean score for clinical patients in the original Green et al. (2008) study. Mean NSP score was 1.98 ± 0.03 (range: 1–7), and mean PRO score was 6.13 ± 0.02 (range: 1–7). As expected, NSP and PRO were negatively correlated (correlation coefficient −0.58, *p* < .001).

### Prediction 1. Paranoia Is Positively Associated With Reward Value of Antisocial Behavior (NSP) and Negatively Associated With Reward Value of Prosocial Behavior (PRO)

**Figure 1 fig1:**
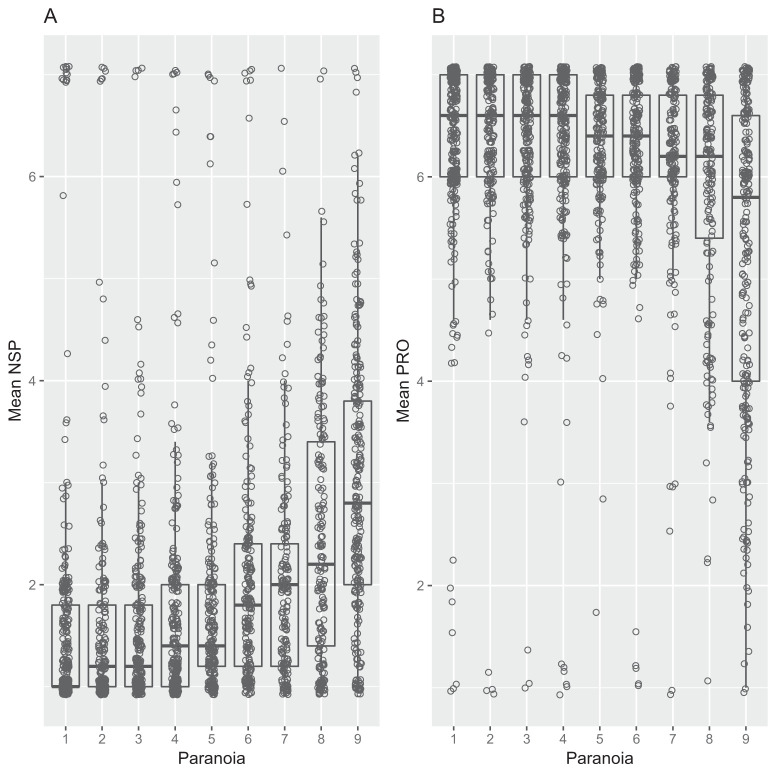
The Association Between Paranoia and (A) Negative Social Potency and (B) Enjoyment of Prosocial Interactions *Note.* Paranoia was converted into a nine-level categorical variable for ease of visualization. Boxplots display the interquartile range with the midline representing the median value. Whiskers are the largest (and smallest) values occurring within 1.5 times of the IQ-range. Raw data are overlaid on top of the boxplots.

Paranoia was positively associated with NSP (estimate, 1.50; 95% CI [1.32, 1.69]; Figure 1a) and negatively associated with PRO (estimate, −1.26; 95% CI [−1.46, −1.06]; Figure 1b). Men had higher NSP (estimate, 0.76; 95% CI [0.58, 0.95]) and lower PRO (estimate, −0.73; 95% CI [−0.95, −0.51]) than women. NSP was negatively associated with age (estimate, −0.36; 95% CI [−0.56, −0.16]) and older people had slightly higher PRO (estimate, 0.04; 95% CI [0.04, 0.52]) than younger people.[Fig-anchor fig1]

### Prediction 2. Paranoia, Harmful Intent Attribution, and NSP Are Associated With Increased Punitive Tendency

**Figure 2 fig2:**
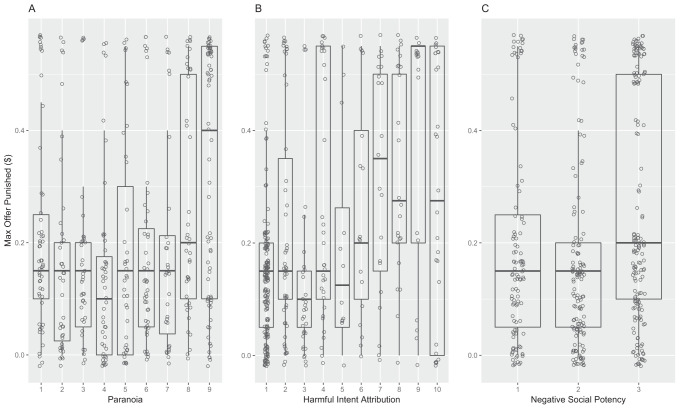
Maximum Offer Punished as a Function of (A) Paranoia, (B) Harmful Intent Attribution, and (C) Negative Social Potency *Note.* For ease of visualization, these plots omit all data points where participants did not punish a partner. Higher maximum offer punished indicates a greater punitive tendency. Raw data are overlaid onto boxplots, with a jitter function. The terms on the *x* axes were converted to categorical variables (paranoia = nine levels, harmful intent attribution = 10 levels, NSP = three levels).

Most people (734 of 1,136, 64.6%) did not punish any dictator offers in this task, although 122 of 1,136 people (10.7%) punished offers of $0.25 or above. Harmful intent attribution and NSP were both positively associated with willingness to punish higher donations (Tables S3 and S4 in the online supplemental materials; Figure 2) and increased total number of offers punished (Table S5 in the online supplemental materials), although we did not detect a main effect of paranoia on either punishment threshold or number of offers punished (see Figure 2).[Fig-anchor fig2]

The failure to detect an independent main effect of paranoia on punishment decisions may have been attributable to the high collinearity between paranoia, and NSP and harmful intent attribution, respectively. This issue was confirmed by the preregistered commonality analysis (Table S4 in the online supplemental materials), which revealed that paranoia uniquely accounted for 6.03% of the explained variance in punishment threshold and 6.58% of variance in total number of offers punished. Together with other terms in the models, paranoia jointly explained a further 30.6% of the variance in each model. Harmful intent attribution had the largest unique explanatory power, accounting for 61.7% of the total variance in punishment threshold that was explained by our model and 60.9% of the variance in total number of offers punished (Table S4 in the online supplemental materials).

### Prediction 4. Paranoia, NSP, and PRO Are Associated With Decreased Generosity in the Dictator Game

**Figure 3 fig3:**
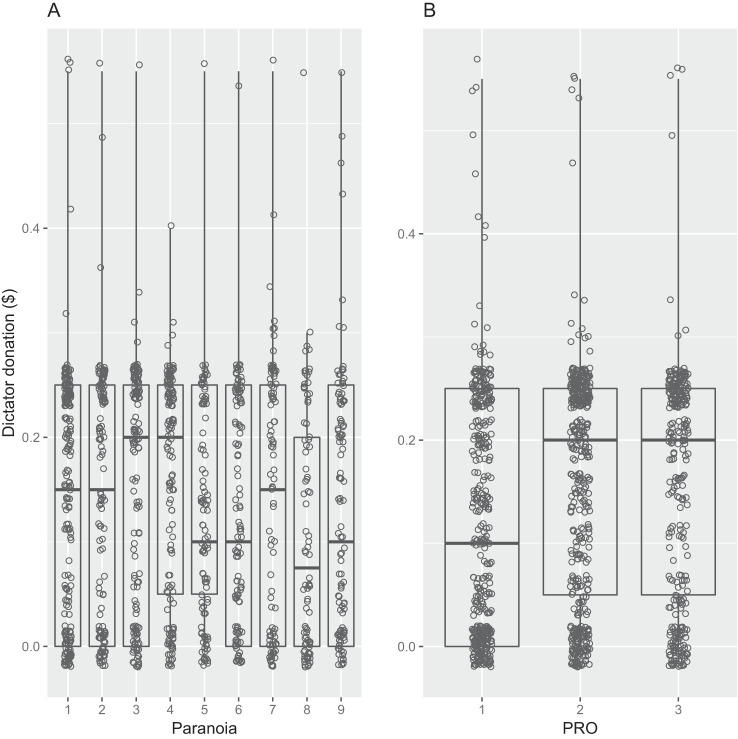
Dictator Donation as a Function of (A) Paranoia and (B) Enjoyment of Prosocial Interactions (PRO) *Note.* Raw data are overlaid onto boxplots, with a jitter function. The terms on the *x* axes were converted to categorical variables (paranoia = nine levels, PRO = three levels).

In the dictator role, participants sent on average $0.14 ± 0.00 (∼25%) of the endowment to their partner. People who had higher PRO scores sent larger amounts to the partner (Table S6 in the online supplemental materials; Figure 3), although there was no additional effect of NSP, probably because of PRO and NSP being strongly negatively correlated. Commonality analysis revealed that NSP uniquely accounted for 2.5% of the explained variance in generosity and 38% when considered in combination with other terms in the model. Counter to our predictions, paranoia had no main effect on generosity in the dictator role (Table S6 in the online supplemental materials; Figure 3). Paranoia uniquely explained 0% of the total variance in dictator donations, although variance common to paranoia and other variables jointly accounted for ∼17% of the variance in dictator donations that was explained by the model (Table S7 in the online supplemental materials). The predictors which uniquely explained most of the variance in dictator donations were age and PRO (Table S7 in the online supplemental materials).[Fig-anchor fig3]

## Discussion

Previous work showed that proneness to paranoid thoughts is positively associated with punitive tendency and negatively associated with generosity in the Dictator Game (Raihani & Bell, 2018). We partially replicate and extend these previous findings here. We show for the first time that paranoid thinking is positively associated with the reward value of negative social behavior and negatively associated with the reward value of positive prosocial interactions. These findings suggest that paranoia is associated with altered patterns of social reward and help to contextualize previous work in this field.

Previous studies have interpreted the reduced prosocial tendency in paranoia in terms of distrust (Ellett et al., 2013; Fett et al., 2012). However, trusting in a trust game can also be construed as a cooperative or prosocial decision (Raihani & Bell, 2018). Previous work exploring alterations to social reward processing in paranoia and psychosis has tended to focus on the reward value of anticipating and/or experiencing cooperation (or not) from a social partner (Fett et al., 2019) despite the fact that social-decisions have also been associated with reward system function independently of response from others (Harbaugh et al., 2007; Moll et al., 2006). The work presented here suggests a role for alteration to intrinsic reward for making social decision in paranoia. Experiencing subjective reward from one’s own prosocial decisions is known as the “warm glow of giving” in the experimental economics literature (Andreoni, 1990), and the reward from making purely altruistic decisions is associated with overlapping, but not identical, reward system activity to strategic giving in fMRI studies (Cutler & Campbell-Meiklejohn, 2019). These common patterns from neuroimaging studies, together with the self-report data we present here, suggest that variation in the reward of prosocial decision-making might be altered in paranoia. Neuroimaging studies in combination with strategic and nonstrategic economic games would be especially useful to confirm this prediction.

Despite being associated, it is important to stress that the causal relationships between paranoia and social reward value are not clear. It is possible that paranoia and altered social reward value share a common cause in some cases—and it is also possible that they mutually reinforce one another (in other words, any causal relationship may not be unidirectional). In this study, we cannot tease apart these possibilities. One common cause of paranoia and reduced NSP (or increased PRO) might be a history of adversity (Howes & Murray, 2014). Exposure to adversity in childhood (including childhood maltreatment, poverty, and low socioeconomic status) is associated with increased tendency to develop paranoia in adulthood (Anderson & Freeman, 2013; Bosqui et al., 2014; Catone et al., 2015; Wickham et al., 2014). Similarly, higher levels of childhood adversity are also implicated in the development of antisocial personality traits, such as lower agreeableness and an increased tendency toward angry feelings (Carver et al., 2014; Labella & Masten, 2018) and appetitive aggression (Dudeck et al., 2016). This suggests one pathway by which paranoia and social reward value might share the same common cause and thus be associated, without paranoia necessarily causing altered social reward value.

We replicate our previous findings that paranoia predicts increased tendency to attribute harmful intent, but not self-interest, to a partner (Raihani & Bell, 2017; Saalfeld et al., 2018). Variation in punitive tendency was largely explained by harmful intent attribution, but negative social potency also played a minor role. Negative social potency was higher among participants who experience a more paranoid thinking style and paranoia in combination with other terms explained around 30% of the variance in punishment decisions. It is possible that the tendency to punish others and proneness to paranoid thinking have a common cause, in that both appear to be associated with exposure to adversity. Herrmann et al. (2008) reported increased antisocial punishment (willingness to punish cooperators) in social environments with low GDP and weak civic norms of cooperation (see also Bone et al., 2016; Raihani & Deutchman, 2017). Similarly, in field-based experimental tasks, exposure to resource scarcity predicted antisocial punishment (Prediger et al., 2014) and increased exploitative strategies (Blanco et al., 2015; Gatiso et al., 2015).

Paranoia was associated with a reduced enjoyment of prosocial behavior but did not explain variation in observed generosity in the Dictator Game. Variance in generosity was largely explained by age and, to a lesser extent, by enjoyment of prosocial interactions. The fact that paranoia was not uniquely associated with variation in generosity contradicts our previous finding (Raihani & Bell, 2018), although we note a potentially important difference between the two studies that might be relevant. In the current study, participants in the dictator role knew that they could be punished by their partner, whereas this information was not supplied in the previous study. The concern about being punished might explain why we fail to detect the negative effect of paranoia on generosity in this study: The association between paranoia and generosity might depend upon strategic concerns. This suggests that paranoia is not simply associated with antisocial tendencies, which would involve both increased punitive behavior and reduced generosity, but that each of these behaviors may be subject to independent risk factors and may fulfill different functions (see also Moll et al., 2018).

There are several caveats and notes of caution when interpreting these data. First, we want to stress that punishment and generosity here are measured in highly abstract settings, involving small sums of money and that an increased willingness to punish in such an experiment should not be taken as indicative of a tendency toward aggression or hostility in real life. Although we recruited a fairly diverse sample, it should also be borne in mind that all data come from U.S.-based participants and that the population on MTurk is younger, less racially diverse, and more politically liberal than the U.S. population average. Ideally, future work would explore the extent to which these findings might generalize across populations and also in samples including patients with a psychotic-spectrum disorder.

## Supplementary Material

10.1037/abn0000647.supp

## References

[c1] Ahmed A. O., Green B. A., Buckley P. F., & McFarland M. E. (2012). Taxometric analyses of paranoid and schizoid personality disorders. Psychiatry Research, 196(1), 123–132. 10.1016/j.psychres.2011.10.010 22377573

[c2] Anderson F., & Freeman D. (2013). Socioeconomic status and paranoia. Journal of Nervous and Mental Disease, 201(8), 698–702. 10.1097/NMD.0b013e31829c5047 23896852

[c3] Andreoni J. (1990). Impure altruism and donations to public goods: A theory of warm-glow giving. Economic Journal, 100(401), 464–477. 10.2307/2234133

[c4] Barnby J. M., Deeley Q., Robinson O., Raihani N., Bell V., & Mehta M. A. (2020). Paranoia, sensitization and social inference: Findings from two large-scale, multi-round behavioural experiments. Royal Society Open Science, 7(3), Article 191525 10.1098/rsos.191525 32269791 PMC7137981

[c5] Bebbington P. E., McBride O., Steel C., Kuipers E., Radovanoviĉ M., Brugha T., Jenkins R., Meltzer H. I., & Freeman D. (2013). The structure of paranoia in the general population. The British Journal of Psychiatry, 202(6), 419–427. 10.1192/bjp.bp.112.119032 23661767

[c6] Blanco E., Lopez M. C., & Villamayor-Tomas S. (2015). Exogenous degradation in the commons: Field experimental evidence. Ecological Economics, 120, 430–439. 10.1016/j.ecolecon.2015.03.028

[c7] Bone J. E., McAuliffe K., & Raihani N. J. (2016). Exploring the motivations for punishment: Framing and country-level effects. PLoS ONE, 11(8), Article e0159769 10.1371/journal.pone.0159769 27487269 PMC4972317

[c8] Bosqui T. J., Hoy K., & Shannon C. (2014). A systematic review and meta-analysis of the ethnic density effect in psychotic disorders. Social Psychiatry and Psychiatric Epidemiology, 49, 519–529. 10.1007/s00127-013-0773-0 24114240

[c9] Brandts J., & Charness G. (2011). The strategy versus the direct-response method: A first survey of experimental comparisons. Experimental Economics, 14, 375–398. 10.1007/s10683-011-9272-x

[c10] Burnham K. P., Anderson D. R., & Huyvaert K. P. (2011). AIC model selection and multimodel inference in behavioral ecology: Some background, observations, and comparisons. Behavioral Ecology and Sociobiology, 65, 23–35. 10.1007/s00265-010-1029-6

[c11] Carver C. S., Johnson S. L., McCullough M. E., Forster D. E., & Joormann J. (2014). Adulthood personality correlates of childhood adversity. Frontiers in Psychology, 5, Article 1357. 25484874 10.3389/fpsyg.2014.01357 PMC4240049

[c12] Catone G., Marwaha S., Kuipers E., Lennox B., Freeman D., Bebbington P., & Broome M. (2015). Bullying victimisation and risk of psychotic phenomena: Analyses of British national survey data. The Lancet Psychiatry, 2(7), 618–624. 10.1016/S2215-0366(15)00055-3 26303559

[c13] Chester D. S. (2017). The role of positive affect in aggression. Current Directions in Psychological Science, 26(4), 366–370. 10.1177/0963721417700457

[c14] Christensen R. H. B. (2018). Cumulative link models for ordinal regression with the R package ordinal. Retrieved from https://cran.r-project.org/web/packages/ordinal/vignettes/clm_article.pdf

[c15] Combs D. R., Penn D. L., Cassisi J., Michael C., Wood T., Wanner J., & Adams S. (2006). Perceived racism as a predictor of paranoia among African Americans. Journal of Black Psychology, 32(1), 87–104. 10.1177/0095798405283175

[c16] Cushman F. A., Dreber A., Wang Y., & Costa J. (2009). Accidental outcomes guide punishment in a “Trembling Hand” game. PLoS ONE, 4(8), Article e6699 10.1371/journal.pone.0006699 19707578 PMC2726629

[c17] Cutler J., & Campbell-Meiklejohn D. (2019). A comparative fMRI meta-analysis of altruistic and strategic decisions to give. NeuroImage, 184, 227–241. 10.1016/j.neuroimage.2018.09.009 30195947

[c18] Diaconescu A. O., Hauke D. J., & Borgwardt S. (2019). Models of persecutory delusions: A mechanistic insight into the early stages of psychosis. Molecular Psychiatry, 24, 1258–1267. 10.1038/s41380-019-0427-z 31076646 PMC6756090

[c19] Dodell-Feder D., Tully L. M., Lincoln S. H., & Hooker C. I. (2014). The neural basis of theory of mind and its relationship to social functioning and social anhedonia in individuals with schizophrenia. NeuroImage Clinical, 4, 154–163. 10.1016/j.nicl.2013.11.006 24371798 PMC3871293

[c20] Dudeck M., Sosic-Vasic Z., Otte S., Rasche K., Leichauer K., Tippelt S., Shenar R., Klingner S., Vasic N., & Streb J. (2016). The association of adverse childhood experiences and appetitive aggression with suicide attempts and violent crimes in male forensic psychiatry inpatients. Psychiatry Research, 240, 352–357. 10.1016/j.psychres.2016.04.073 27138831

[c21] Edens J. F., Marcus D. K., & Morey L. C. (2009). Paranoid personality has a dimensional latent structure: Taxometric analyses of community and clinical samples. Journal of Abnormal Psychology, 118(3), 545–553. 10.1037/a0016313 19685951 PMC2853919

[c22] Elahi A., Perez Algorta G., Varese F., McIntyre J. C., & Bentall R. P. (2017). Do paranoid delusions exist on a continuum with subclinical paranoia? A multi-method taxometric study. Schizophrenia Research, 190, 77–81. 10.1016/j.schres.2017.03.022 28318838

[c23] Ellett L., Allen-Crooks R., Stevens A., Wildschut T., & Chadwick P. (2013). A paradigm for the study of paranoia in the general population: The Prisoner’s Dilemma Game. Cognition and Emotion, 27(1), 53–62. 10.1080/02699931.2012.689757 22731988

[c24] Fett A.-K. J., Mouchlianitis E., Gromann P. M., Vanes L., Shergill S. S., & Krabbendam L. (2019). The neural mechanisms of social reward in early psychosis. Social Cognitive and Affective Neuroscience, 14(8), 861–870. 10.1093/scan/nsz058 31506672 PMC6847053

[c25] Fett A., Shergill S. S., Joyce D. W., Riedl A., Strobel M., Gromann P. M., & Krabbendam L. (2012). To trust or not to trust: The dynamics of social interaction in psychosis. Brain: A Journal of Neurology, 135(3), 976–984. 10.1093/brain/awr359 22366802

[c26] Fischbacher U., Gachter S., & Quercia S. (2012). The behavioral validity of the strategy method in public good experiments. Journal of Economic Psychology, 33(4), 897–913. 10.1016/j.joep.2012.04.002

[c27] Foulkes L. (2019). Sadism: Review of an elusive construct. Personality and Individual Differences, 151, Article 109500 10.1016/j.paid.2019.07.010

[c28] Foulkes L., Viding E., McCrory E., & Neumann C. S. (2014). Social Reward Questionnaire (SRQ): Development and validation. Frontiers in Psychology, 5, Article 289 10.3389/fpsyg.2014.00201 24653711 PMC3949132

[c29] Freeman D., & Fowler D. (2009). Routes to psychotic symptoms: Trauma, anxiety and psychosis-like experiences. Psychiatry Research, 169(2), 107–112. 10.1016/j.psychres.2008.07.009 19700201 PMC2748122

[c30] Freeman D., & Garety P. A. (2000). Comments on the content of persecutory delusions: Does the definition need clarification? British Journal of Clinical Psychology, 39, 407–414. 10.1348/014466500163400 11107494

[c31] Freeman D., Garety P. A., Bebbington P. E., Smith B., Rollinson R., Fowler D., Kuipers E., Ray K., & Dunn G. (2005). Psychological investigation of the structure of paranoia in a non-clinical population. The British Journal of Psychiatry, 186(5), 427–435. 10.1192/bjp.186.5.427 15863749

[c32] Freeman P. D. (2016). Persecutory delusions: A cognitive perspective on understanding and treatment. The Lancet Psychiatry, 3(7), 685–692. 10.1016/S2215-0366(16)00066-3 27371990

[c33] Gatiso T. T., Vollan B., & Nuppenau E.-A. (2015). Resource scarcity and democratic elections in commons dilemmas: An experiment on forest use in Ethiopia. Ecological Economics, 114, 199–207. 10.1016/j.ecolecon.2015.04.005

[c34] Gelman A. (2008). Scaling regression inputs by dividing by two standard deviations. Statistics in Medicine, 27(15), 2865–2873. 10.1002/sim.3107 17960576

[c35] Gerstenberg T., Lagnado D. A., & Kareev Y. (2010). The dice are cast: The role of intended versus actual contributions in responsibility attribution In Ohlsson S. & Catrambone R. (Eds.), Proceedings of the Annual Meeting of the Cognitive Science Society (Vol. 32, pp. 1697–1702). Cognitive Science Society.

[c36] Green C. E. L., Freeman D., Kuipers E., Bebbington P., Fowler D., Dunn G., & Garety P. A. (2008). Measuring ideas of persecution and social reference: The Green et al. Paranoid Thought Scales (GPTS). Psychological Medicine. 38(1), 101–111. 17903336 10.1017/S0033291707001638

[c37] Greenburgh A., Bell V., & Raihani N. J. (2019). Paranoia and conspiracy: Group cohesion increases harmful intent attribution in the Trust Game. PeerJ, 7, Article e7403 10.7717/peerj.7403 31440431 PMC6699476

[c38] Gromann P. M., Heslenfeld D. J., Fett A.-K., Joyce D. W., Shergill S. S., & Krabbendam L. (2013). Trust versus paranoia: Abnormal response to social reward in psychotic illness. Brain: A Journal of Neurology, 136(6), 1968–1975. 10.1093/brain/awt076 23611807

[c39] Gromann P. M., Shergill S. S., de Haan L., Meewis D. G. J., Fett A.-K. J., Korver-Nieberg N., & Krabbendam L. (2014). Reduced brain reward response during cooperation in first-degree relatives of patients with psychosis: An fMRI study. Psychological Medicine, 44(16), 3445–3454. 10.1017/S0033291714000737 25065732

[c40] Grueber C. E., Nakagawa S., Laws R. J., & Jamieson I. G. (2011). Multimodel inference in ecology and evolution: Challenges and solutions. Journal of Evolutionary Biology, 24(4), 699–711. 10.1111/j.1420-9101.2010.02210.x 21272107

[c41] Hanewald B., Behrens F., Gruppe H., Sammer G., Gallhofer B., Krach S., & Iffland J. R. (2017). Anticipation of social and monetary rewards in schizophrenia. Journal of Psychiatry, 20(3), 1–7. 10.4172/2378-5756.1000410

[c42] Hanssen E., Fett A.-K., White T. P., Caddy C., Reimers S., & Shergill S. S. (2018). Cooperation and sensitivity to social feedback during group interactions in schizophrenia. Schizophrenia Research, 202, 361–368. 10.1016/j.schres.2018.06.065 30005931

[c43] Harbaugh W. T., Mayr U., & Burghart D. R. (2007). Neural responses to taxation and voluntary giving reveal motives for charitable donations. Science, 316(5831), 1622–1625. 10.1126/science.1140738 17569866

[c44] Herrmann B., Thöni C., & Gachter S. (2008). Antisocial punishment across societies. Science, 319(5868), 1362–1367. 10.1126/science.1153808 18323447

[c45] Howes O. D., & Murray R. M. (2014). Schizophrenia: An integrated sociodevelopmental-cognitive model. The Lancet, 383(9929), 1677–1687. 10.1016/S0140-6736(13)62036-X PMC4127444 24315522

[c46] Kahneman D., Knetsch J. L., & Thaler R. (1986). Fairness as a constraint on profit seeking: Entitlements in the market. The American Economic Review, 76(4), 728–741.

[c47] Labella M. H., & Masten A. S. (2018). Family influences on the development of aggression and violence. Current Opinion in Psychology, 19, 11–16. 10.1016/j.copsyc.2017.03.028 29279207

[c48] Leach C. W., Spears R., Branscombe N. R., & Doosje B. (2003). Malicious pleasure: Schadenfreude at the suffering of another group. Journal of Personality and Social Psychology, 84(5), 932–943. 10.1037/0022-3514.84.5.932 12757139

[c49] Lemmers-Jansen I. L. J., Fett A.-K. J., Hanssen E., Veltman D. J., & Krabbendam L. (2019). Learning to trust: Social feedback normalizes trust behavior in first-episode psychosis and clinical high risk. Psychological Medicine, 49(5), 780–790. 10.1017/S003329171800140X 29897026

[c50] Moll J., de Oliveira-Souza R., Basilio R., Bramati I. E., Gordon B., Rodríguez-Nieto G., Zahn R., Krueger F., & Grafman J. (2018). Altruistic decisions following penetrating traumatic brain injury. Brain: A Journal of Neurology, 141(5), 1558–1569. 10.1093/brain/awy064 29590314 PMC7341482

[c51] Moll J., Krueger F., Zahn R., Pardini M., de Oliveira-Souza R., & Grafman J. (2006). Human fronto–mesolimbic networks guide decisions about charitable donation. Proceedings of the National Academy of Sciences of the United States of America, 103(42), 15623–15628. 10.1073/pnas.0604475103 17030808 PMC1622872

[c52] Nimon K., Oswald F., & Roberts J. K. (2020). yhat: Interpreting regression effects (R package version 2) [Computer software]. Retrieved from https://cran.r-project.org/web/packages/yhat/yhat.pdf

[c53] Nimon K., & Reio T. G. Jr. (2011). Regression Commonality Analysis: A Technique for Quantitative Theory Building. Human Resource Development Review, 10(3), 329–340. 10.1177/1534484311411077

[c54] O’Meara A., Davies J., & Hammond S. (2011). The psychometric properties and utility of the Short Sadistic Impulse Scale (SSIS). Psychological Assessment, 23(2), 523–531. 10.1037/a0022400 21319907

[c55] Prediger S., Vollan B., & Herrmann B. (2014). Resource scarcity and antisocial behavior. Journal of Public Economics, 119, 1–9. 10.1016/j.jpubeco.2014.07.007

[c56] Raihani N. J., & Bell V. (2018). Conflict and cooperation in paranoia: A large-scale behavioural experiment. Psychological Medicine, 48(9), 1523–1531. 29039293 10.1017/S0033291717003075 PMC6088528

[c57] Raihani N. J., & Bell V. (2017). Paranoia and the social representation of others: A large-scale game theory approach. Scientific Reports, 7, Article 4544 10.1038/s41598-017-04805-3 28674445 PMC5495777

[c58] Raihani N. J., & Bell V. (2019). An evolutionary perspective on paranoia. Nature Human Behaviour, 3, 114–121. 10.1038/s41562-018-0495-0 PMC6420131 30886903

[c59] Raihani N. J., & Deutchman P. (2017). Dark Triad personality traits vary across countries and predict antisocial behavior. PsyArXiv Retrieved from https://psyarxiv.com/8t6k5/

[c60] Ray Mukherjee J., Nimon K., Mukherjee S., Morris D. W., Slotow R., & Hamer M. (2014). Using commonality analysis in multiple regressions: A tool to decompose regression effects in the face of multicollinearity. Methods in Ecology and Evolution, 5(4), 320–328. 10.1111/2041-210X.12166

[c61] Ritsner M. S., Ratner Y., Mendyk N., & Gooding D. C. (2018). The characterization of social anhedonia and its correlates in schizophrenia and schizoaffective patients. Psychiatry Research, 270, 922–928. 10.1016/j.psychres.2018.11.003 30551345

[c62] Saalfeld V., Ramadan Z., Bell V., & Raihani N. J. (2018). Experimentally induced social threat increases paranoid thinking. Royal Society Open Science, 5(8), Article 180569 10.1098/rsos.180569 30225050 PMC6124070

[c63] Sarin A., Lagnado D. A., & Burgess P. W. (2017). The intention-outcome asymmetry effect. Experimental Psychology, 64(2), 124–141. 10.1027/1618-3169/a000359 28497723

[c64] Schaechtele S., Gerstenberg T., & Lagnado D. (2011). Beyond outcomes: The influence of intentions and deception Proceedings of the 33rd Annual Conference of the Cognitive Science Society (pp. 1860–1865). Cognitive Science Society.

[c65] Wickham S., Taylor P., Shevlin M., & Bentall R. P. (2014). The impact of social deprivation on paranoia, hallucinations, mania and depression: The role of discrimination social support, stress and trust. PLoS ONE, 9(8), Article e105140 10.1371/journal.pone.0105140 25162703 PMC4146475

