# Supplementary material for: Social Reward, Punishment, and Prosociality in Paranoia
Source: J Abnorm Psychol. 2020 Dec 3;130(2):177–85. doi: 10.1037/abn0000647 (PMC7832736; doi:10.1037/abn0000647)
Supplement: Supplementary file 1 [file ABN-2020-1504_Suppl.docx]

**Social reward, punishment and prosociality in paranoia – Supplementary Information**

Nichola Raihani, Daniel Martinez-Gatell, Vaughan Bell & Lucy Foulkes

Contents

1. Description of the Green et al. Paranoid Thought Scales
2. Description of the Social Reward Questionnaire
3. Procedure
4. Statistical methods
5. Measuring intention attribution in the Dictator Game
6. Tables S1-S7
7. **Description of the Green et al. Paranoid Thought Scales**

The Green et al. Paranoid Thought Scales (2008) is an instrument comprised of two subscales which measure feelings of social reference (e.g. “I spent time thinking about friends gossiping about me”) and ideas of persecution (e.g. “Certain individuals have had it in for me”), respectively. Participants rated on a Likert Scale (1-5) the extent to which they agreed with 16 statements in each subscale. Paranoia scores for each participant were obtained by summing the scores from these two subscales, with the minimum score being 32 and the maximum being 160. Higher scores indicate higher levels of paranoia. The average score for patients with a clinical diagnosis of a psychotic spectrum disorder is 101.9 and Green et al. (2008) reported around 5 % of people in the general population to score above this clinical mean.

1. **Description of the Social Reward Questionnaire**

The Social Reward Questionnaire (Foulkes et al., 2014) is a collection of six subscales that respectively measure individual differences in the hedonic value of different types of social interactions. Since the subscales do not map onto a single higher-order factor of social reward, researchers can use as many or as few of the subscales as they wish in their research. All subscales of the SRQ are reliable, valid and internally consistent, and associate with related external factors (Foulkes et al., 2014). We had specific a priori predictions about the Negative Social Potency and Prosocial Interactions subscales (hereafter NSP and PRO, respectively) and, as such, participants in our study only took these two subscales. Each consisted of five items where participants rate on a Likert scale (1-7) the extent to which they agree or disagree with the statement.

Negative social potency is the subjective reward value a person reports experiencing from engaging in harmful or callous behaviour. The capacity to experience reward from other people’s pain, suffering or misfortune is prevalent across community and clinical samples (Foulkes, 2019). In addition to negative social potency (Foulkes et al., 2014), this phenomenon of enjoying causing harm has been identified as appetitive aggression (Chester, 2017), sadism (e.g. (O’Meara et al., 2011)) and schadenfreude (e.g. (Leach et al., 2003)). We expected that paranoia would be associated with increased negative social potency.

We also expected that paranoia would be negatively associated with the propensity to experience reward from positive, prosocial interactions, which is another common form of subjective social reward (Foulkes et al., 2014). Aberrant subjective reward processing might account for empirical findings showing that people with psychosis are less likely to trust a social interaction partner (Fett et al., 2012; Gromann et al., 2013), and more recent work showing that paranoia is associated with a general reduction in generosity in social interactions, even when trust is not a strategic concern (Raihani and Bell, 2017a). Evidence from patients with schizophrenia also suggests variation in social reward processing, with patients showing reduced responsiveness to social rewards (smiling faces, (Hanewald et al., 2017)) and increased self-reported social anhedonia (Dodell-Feder et al., 2014; Ritsner et al., 2018).

1. **Procedure**

We maintained a minimum interval of seven days between the psychometric instruments and recalling participants for the first experimental task, to minimise behavioural spillover from the survey to the task. When recalled, participants were invited to take part in a modified Dictator Game (described below). Participants were allocated either to the role of receiver or dictator (within-subject, order counter-balanced). After a minimum interval of 10 days, participants were recalled again and assigned to the opposite role. Of the original 2,004 participants, we successfully re-recruited and obtained full responses from 1138 / 2004 participants in the dictator role and 1136 / 2004 participants in the receiver role.

The Dictator Game is a two-player game where one person, the dictator, is endowed with a sum of money and can choose how much to send to the partner, the receiver (Kahneman et al., 1986). In the standard Dictator Game, the dictator can send however much they want to the receiver and the receiver simply has to accept whatever the dictator sends. Our Dictator Game was modified to allow receivers to pay to punish their partners after seeing how much the partner decided to send (see below), thereby allowing us to measure punitive tendency. Note that while loaded terms such as dictator, punish, fair and unfair are used to describe the task below, we did not use these terms in the instructions seen by participants. All materials are available online at <https://osf.io/6kv3d/>.

*Measuring punitive tendency and intention attribution*

We measured (i) the attribution of harmful intent and self-interest; and (ii) punitive tendency by casting participants in the receiver role in two separate Dictator Games (with different partners, order counter-balanced). We refer to these as Game A and Game B henceforth. In each game, the receiver was initially endowed with $0.05 and knew that their partner, the dictator, was endowed with $0.55. In Game A, we measured harmful intent attribution. We presented receivers with the outcome of a binary decision (fair / unfair) as we were simply interested in how the tendency to attribute harmful intent in a fair/unfair setting would affect the punishment decision in Game B (see predictions below). The receiver was told that the dictator could choose whether to send them $0.00 (unfair) or to send them $0.25 (fair). If dictators chose the fair option, then both players ended up with the same amount: $0.30. If dictators chose the unfair option, then the dictator kept $0.55 and the receiver kept their initial $0.05. After being presented with either a fair or unfair decision, receivers were then asked to rate on two separate slider scales (0-100, where 0 = not at all and 100 = completely) the extent to which they believed dictator decisions were motivated by ‘a desire to earn more money’ (hereafter, self-interest) or by ‘a desire to reduce your bonus’ (hereafter, harmful intent).

In Game B, dictators and receivers had the same starting endowments but dictators could send any amount (in $0.05 increments) to the receiver. Receivers stated for each possible donation from the dictator whether they would choose to punish the partner. Game B therefore allowed us to attempt to replicate a previously reported result showing that paranoia predicts increased willingness to punish partners in social interactions (Raihani and Bell, 2017a) and to ask to what extent harmful intent attribution and NSP also contributed to the willingness to punish a partner.

*Measuring generosity*

In a separate wave of data collection, all participants were also assigned to the role of ‘dictator’ in these games, so that we could measure variation in generosity. The order in which participants were assigned to receiver or dictator roles was counter-balanced. Participants were allocated to the roles of dictator in Game A and Game B (described above) but we only analysed data from Game B (where participants could send any amount from the $0.55, rather than making a binary choice to share or not with the partner). This analysis decision was pre-registered. Because we were not interested in behavioural data from Game A (this was collected so that we could truthfully inform receivers in this game that they were responding to decisions made by real people) we did not counter-balance the order in which people played in Game A and Game B when they were in the dictator role.

2.2.4 Measuring generosity

Participants were cast in the dictator role in two Dictator Games (Game C and Game D), with two different participants. Only data from Game C were analysed (as per the pre-registered predictions) and task order was therefore not counter-balanced across participants. Game D data were collected only so that we could truthfully inform participants in the receiver role (in Game A) that they were responding to decisions made by real workers.

*1. Paranoia is associated with increased tendency to attribute harmful intentions, but not increased self-interest, to partner in the dictator game.*

We set harmful intent attribution and self-interest attribution as ordinal categorical response terms (each with 10 levels) in two clms, with paranoia, dictator offer (fair/unfair), age, comprehension, gender and order as explanatory terms in each model. Paranoia positively predicted harmful intent attribution (estimate: 0.84, CI: 0.60, 1.08, Table S1) but not attributions of self-interest (paranoia was not a component of the top model set predicting variation in self-interest attribution). In an unregistered analysis, we also found that, on average, people were more likely to attribute self-interest than harmful intent to their interaction partners (mean self-interest: 64.2 ± 1.13, mean harmful intent: 18.5 ± 0.83; paired t test, *t* = -36.7, df = 1135, *p* < 0.001). As expected, people attributed less harmful intent (estimate: -0.69, CI: -0.92, -0.45) and less self-interest (estimate: -4.07, CI: -4.40, -3.74) when they played against fair (compared to unfair) dictators (Tables S1 & S2).

1. **Statistical Methods**

All data were analysed using R package 3.5.0. Models were constructed as cumulative link models (clm) using the using the R package ordinal (Christensen, 2018). Cumulative link models allow ordinal categorical variables to be specified as response terms. Explanatory terms in the models were specified a priori and pre-registered (see https://osf.io/6kv3d/). Continuous explanatory terms were standardized (by dividing by two standard deviations) and binary input variables were centered (Gelman, 2008). Effect sizes and confidence intervals for all analyses were calculated using multi-model selection with full model averaging (which provides conservative estimates). This approach has been described extensively elsewhere (Burnham et al., 2011; Grueber et al., 2011).

For models based on the dictator or receiver behaviour, we also included a binary term for comprehension, which was set to 1 if the participant answered all comprehension questions correctly and 0 otherwise. In the receiver task, 130 / 1136 (11.4 %) participants failed at least one comprehension check. In the dictator task, 174 / 1138 (15.2 %) participants failed at least one comprehension check. For models based on receiver behaviour, we also included a term for order, which denoted whether the participant took part in the intention attribution task (Game A) or the punishment task (Game B) first.

We used the R package yhat to perform commonality analyses. This allows one to determine how much of the explained variance in a dependent variable is uniquely explained by a given predictor variable, and how much of the explained variance is common to multiple predictors (Nimon and Reio, 2011). Specifically, commonality analysis allows one to partition coefficients into unique and common effects – a technique that is particularly helpful when models contain correlated predictor variables (Ray Mukherjee et al., 2014).

2.3.2 Deviations from planned analyses

(i) We originally planned to perform mediation analyses to explore whether variables that correlate with paranoia (harmful intent, NSP, PRO) mediate paranoia’s association with (i) punitive tendency and (ii) generosity, respectively. However, we latterly realised that this approach was not appropriate for two main reasons. First, paranoia was not a randomly-assigned variable (so determining that paranoia *causes* NSP / PRO, rather than simply being associated with them, was not possible). Second, we believe that there is a high chance that paranoia and personality factors (like NSP and PRO) have a common cause in an unmeasured third variable. For example, a history of trauma predicts higher levels of both paranoia and appetitive aggression (e.g. (Dudeck et al., 2016; Freeman and Fowler, 2009)) suggesting increased negative social potency and paranoia may be related through joint aetiology. Without the ability to measure this third variable, the mediation analysis would be confounded. For these reasons, we do not report the mediation analyses that we originally pre-registered for this study.

(ii) We report a deviation from the pre-registration that affects the punishment analyses only. After we had finished collecting data, we noticed a problem with the study design: measures of harmful intent attribution for each participant will depend on whether that participant was responding to a fair or unfair offer in Game A (with harmful intent attributions being stronger when interacting with an unfair, rather than a fair, partner (Raihani and Bell, 2017b)). Including raw measures of harmful intent attribution to explain punishment decisions in Game B is therefore incorrect because harmful intent measures were affected by the treatment arm the participant was assigned to in Game A. To account for this, we created a variable called ‘residual Harmful Intent’, which is the residual variance in harmful intent attribution once the effect of interacting with a fair or unfair dictator has been accounted for. This variable was created by running a regression analysis of fairness (dictator fair / dictator unfair) on harmful intent attribution and taking the residuals from the model. We use this residual variable in the analyses rather than the uncorrected measures of harmful intent attribution.

1. **Measuring intention attribution in the Dictator Game**

We measured (i) the attribution of harmful intent and self-interest; and (ii) punitive tendency by casting participants in the receiver role in two separate Dictator Games (with different partners, order counter-balanced). In each game, the receiver was initially endowed with $0.05 and knew that their partner, the dictator, was endowed with $0.55. In Game A, we measured harmful intent attribution. The receiver was told that the dictator could choose whether to send them $0.00 (unfair) or to send them $0.25 (fair). If dictators chose the fair option, then both players ended up with the same amount: $0.30. If dictators chose the unfair option, then the dictator kept $0.55 and the receiver kept their initial $0.05. After being presented with either a fair or unfair decision, receivers were then asked to rate on two separate slider scales (0-100, where 0 = not at all and 100 = completely) the extent to which they believed dictator decisions were motivated by ‘a desire to earn more money’ (hereafter, self-interest) or by ‘a desire to reduce your bonus’ (hereafter, harmful intent).

*1. Paranoia is associated with increased tendency to attribute harmful intentions, but not increased self-interest, to partner in the dictator game.*

We set harmful intent attribution and self-interest attribution as ordinal categorical response terms (each with 10 levels) in two clms, with paranoia, dictator offer (fair/unfair), age, comprehension, gender and order as explanatory terms in each model. Paranoia positively predicted harmful intent attribution (estimate: 0.84, CI: 0.60, 1.08, Table S1) but not attributions of self-interest (paranoia was not a component of the top model set predicting variation in self-interest attribution). In an unregistered analysis, we also found that, on average, people were more likely to attribute self-interest than harmful intent to their interaction partners (mean self-interest: 64.2 ± 1.13, mean harmful intent: 18.5 ± 0.83; paired t test, *t* = -36.7, df = 1135, *p* < 0.001). As expected, people attributed less harmful intent (estimate: -0.69, CI: -0.92, -0.45) and less self-interest (estimate: -4.07, CI: -4.40, -3.74) when they played against fair (compared to unfair) dictators (Tables S1 & S2).

1. **Table S1. Variables associated with attributions of harmful intent.**

| **Parameter** | **Estimate** | **Unconditional**  **SE** | **Confidence Interval** | **Relative Importance** |
| --- | --- | --- | --- | --- |
| *Intercept 1\|2* | *1.24* | *0.18* | *(0.88, 1.60)* |  |
| *Intercept 2\|3* | *1.84* | *0.19* | *(1.47, 2.21)* |  |
| *Intercept 3\|4* | *2.25* | *0.19* | *(1.87, 2.63)* |  |
| *Intercept 4\|5* | *2.66* | *0.20* | *(2.26, 3.05)* |  |
| *Intercept 5\|6* | 2.81 | 0.20 | *(2.41, 3.21)* |  |
| *Intercept 6\|7* | 3.12 | 0.21 | *(2.71, 3.52)* |  |
| *Intercept 7\|8* | 3.48 | 0.22 | *(3.06, 3.90)* |  |
| *Intercept 8\|9* | 3.90 | 0.23 | *(3.45, 4.34)* |  |
| *Intercept 9\|10* | 4.35 | 0.24 | *(3.88, 4.83)* |  |
| Comprehension (1=full comprehension) | -1.44 | 0.18 | (-1.80, -1.08) | 1.00 |
| Paranoia | 0.84 | 0.12 | (0.60, 1.08) | 1.00 |
| Fairness (1=partner fair) | -0.69 | 0.12 | (-0.92, -0.45) | 1.00 |
| Male | -0.29 | 0.12 | (-0.53, -0.05) | 1.00 |
| Order | 0.17 | 0.14 | (-0.11, 0.45) | 0.74 |
| Age | -0.01 | 0.06 | (-0.13, 0.11) | 0.21 |

Harmful intent was coded as a ten-level ordinal categorical variable and set as the response term in a clm (Christensen 2016). Importance is the probability that the term in question is a component of the true best model.

1. **Table S2. Variables associated with attributions of self-interest.**

| **Parameter** | **Estimate** | **Unconditional**  **SE** | **Confidence Interval** | **Relative Importance** |
| --- | --- | --- | --- | --- |
| *Intercept 1\|2* | *-3.09* | *0.21* | *(-3.49, -2.68)* |  |
| *Intercept 2\|3* | *-2.34* | *0.20* | *(-2.74, -1.95)* |  |
| *Intercept 3\|4* | *-1.89* | *0.20* | *(-2.28, -1.50)* |  |
| *Intercept 4\|5* | *-1.60* | *0.20* | *(-1.99, -1.22)* |  |
| *Intercept 5\|6* | -1.44 | 0.20 | *(-1.82, -1.06)* |  |
| *Intercept 6\|7* | -1.10 | 0.19 | *(-1.48, -0.72)* |  |
| *Intercept 7\|8* | -0.63 | 0.19 | *(-1.01, -0.25)* |  |
| *Intercept 8\|9* | -0.08 | 0.19 | *(-0.46, 0.30)* |  |
| *Intercept 9\|10* | 0.53 | 0.19 | *(0.15, 0.91)* |  |
| Fairness | -4.07 | 0.17 | (-4.40, -3.74) | 1.00 |
| Age | -0.06 | 0.11 | (-0.28, 0.15) | 0.40 |
| Comprehension | -0.06 | 0.14 | (-0.34, 0.22) | 0.31 |
| Male | 0.00 | 0.04 | (-0.09, 0.08) | 0.12 |

Self-interest was coded as a ten-level ordinal categorical variable and set as the response term in a clm (Christensen 2016). Importance is the probability that the term in question is a component of the true best model.

**Table S3. Variables associated with highest offer punished (punishment threshold).**

| **Parameter** | **Estimate** | **Unconditional**  **SE** | **Confidence Interval** | **Relative Importance** |
| --- | --- | --- | --- | --- |
| *Intercept 1\|2* | *1.00* | *0.22* | *(0.58, 1.42)* |  |
| *Intercept 2\|3* | *1.32* | *0.22* | *(0.89, 1.75)* |  |
| *Intercept 3\|4* | *1.56* | *0.22* | *(1.12, 1.99)* |  |
| *Intercept 4\|5* | *1.83* | *0.22* | *(1.40, 2.27)* |  |
| *Intercept 5\|6* | 2.33 | 0.23 | *(1.89, 2.78)* |  |
| *Intercept 6\|7* | 2.80 | 0.24 | *(2.33, 3.26)* |  |
| *Intercept 7\|8* | 3.29 | 0.25 | *(2.80, 3.78)* |  |
| *Intercept 8\|9* | 3.65 | 0.26 | *(3.14, 4.16)* |  |
| Comprehension | -1.38 | 0.19 | (-1.76, -1.01) | 1.00 |
| Harmful Intent | 1.05 | 0.13 | (0.80, 1.30) | 1.00 |
| NSP | 0.33 | 0.13 | (0.08, 0.58) | 1.00 |
| Order | 0.16 | 0.15 | (-0.13, 0.44) | 0.72 |
| Male | 0.16 | 0.15 | (-0.13, 0.45) | 0.70 |
| Paranoia | 0.14 | 0.15 | (-0.16, 0.45) | 0.63 |
| Age | -0.02 | 0.07 | (-0.15, 0.11) | 0.16 |

Punishment threshold was coded as a nine-level ordinal categorical variable and set as the response term in a clm (Christensen 2016). Higher thresholds indicate increased willingness to punish.

**Table S4. Commonality analyse for punishment threshold and number of offers punished.**

|  | **Punishment threshold** | | **Offers Punished** | |
| --- | --- | --- | --- | --- |
| **Parameter** | **Coefficient** | **% Variance** | **Coefficient** | **% Variance** |
| Paranoia | 9.64 | 6.03 | 10.5 | 6.58 |
| NSP | 9.51 | 5.95 | 10.9 | 6.84 |
| Harmful Intent | 98.6 | 61.7 | 96.7 | 60.9 |
| Paranoia & NSP | 8.03 | 5.02 | 8.90 | 5.60 |
| Paranoia & Harmful Intent | 21.2 | 13.3 | 20.0 | 12.6 |
| NSP & Harmful Intent | 2.90 | 2.81 | 2.68 | 1.69 |
| Paranoia & NSP & Harmful Intent | 9.92 | 6.21 | 9.22 | 5.80 |
|  |  | |  | |

Commonality analysis showing the coefficients and percentage of the total variance in (i) punishment threshold and (ii) number of offers punished explained by each term uniquely and in combination with other terms.

**Table S5.** Variables associated with number of offers punished.

| **Parameter** | **Estimate** | **Unconditional**  **SE** | **Confidence Interval** | **Relative Importance** |
| --- | --- | --- | --- | --- |
| *Intercept 1\|2* | *1.01* | *0.25* | *(0.53, 1.50)* |  |
| *Intercept 2\|3* | *1.39* | *0.25* | *(0.90, 1.88)* |  |
| *Intercept 3\|4* | *1.67* | *0.25* | *(1.17, 2.16)* |  |
| *Intercept 4\|5* | *2.01* | *0.25* | *(1.51, 2.51)* |  |
| *Intercept 5\|6* | 2.71 | 0.26 | *(2.19, 3.22)* |  |
| *Intercept 6\|7* | 3.33 | 0.28 | *(2.79, 3.88)* |  |
| *Intercept 7\|8* | 3.90 | 0.29 | *(3.33, 4.47)* |  |
| Comprehension | -1.38 | 0.19 | (-1.76, -1.01) | 1.00 |
| Harmful Intent | 1.05 | 0.13 | (0.80, 1.31) | 1.00 |
| NSP | 0.35 | 0.13 | (0.10, 0.61) | 1.00 |
| Male | 0.23 | 0.15 | (-0.06, 0.51) | 0.89 |
| Order | 0.16 | 0.14 | (-0.12, 0.44) | 0.74 |
| Paranoia | 0.16 | 0.16 | (-0.15, 0.47) | 0.67 |
| Age | -0.03 | 0.09 | (-0.21, 0.14) | 0.24 |

Number of offers punished was coded as an eight-level ordinal categorical variable and set as the response term in a clm (Christensen 2016).

**Table S6. Variables associated with donation size in the Dictator Game.**

| **Parameter** | **Estimate** | **Unconditional**  **SE** | **Confidence Interval** | **Relative Importance** |
| --- | --- | --- | --- | --- |
| *Intercept 1\|2* | *1.67* | *0.47* | *(0.75, 2.59)* |  |
| *Intercept 2\|3* | *1.97* | *0.47* | *(1.05, 2.89)* |  |
| *Intercept 3\|4* | *2.35* | *0.47* | *(1.42, 3.27)* |  |
| *Intercept 4\|5* | *2.87* | *0.47* | *(1.94, 3.80)* |  |
| *Intercept 5\|6* | 5.62 | 0.50 | *(4.63, 6.61)* |  |
| PRO | 0.50 | 0.13 | (0.24, 0.76) | 1.00 |
| Age | 0.45 | 0.11 | (0.23, 0.67) | 1.00 |
| NSP | -0.04 | 0.10 | (-0.23, 0.15) | 0.25 |
| Paranoia | -0.01 | 0.05 | (-0.10, 0.09) | 0.14 |
| Comprehension | 0.01 | 0.06 | (-0.11, 0.12) | 0.13 |
| Male | 0.01 | 0.04 | (-0.08, 0.09) | 0.13 |

Donation was coded as a six-level ordinal categorical variable and set as the response term in a clm (Christensen 2016).

**Table S7. Commonality analysis for variables associated with dictator donation.**

|  | **Dictator donation** | |
| --- | --- | --- |
| **Parameter** | **Coefficient** | **% Variance** |
| Paranoia | 0.03 | 0.00 |
| NSP | 1.18 | 2.48 |
| PRO | 9.34 | 19.7 |
| Age | 15.4 | 32.4 |
| Paranoia & NSP | 0.21 | 0.05 |
| Paranoia & PRO | 0.19 | 0.04 |
| NSP & PRO | 8.54 | 18.0 |
| Paranoia & Age | 0.74 | 1.56 |
| NSP & Age | 0.41 | 1.00 |
| PRO & Age | 1.70 | 3.59 |
| Paranoia & NSP & PRO | 2.57 | 5.40 |
| Paranoia & NSP & Age | 0.74 | 1.55 |
| Paranoia & PRO & Age | 0.58 | 1.22 |
| NSP & PRO & Age | 2.47 | 5.21 |
| Paranoia & NSP & PRO & Age | 3.35 | 7.06 |
|  |  | |

Commonality analysis showing the coefficients and percentage of the total variance in dictator donation explained by each term uniquely and in combination with other terms.

**References**

Burnham, K.P., Anderson, D.R., and Huyvaert, K.P. (2011). AIC model selection and multimodel inference in behavioral ecology: some background, observations, and comparisons. Behav Ecol Sociobiol *65*, 23–35.

Chester, D.S. (2017). The Role of Positive Affect in Aggression. Curr Dir Psychol Sci *26*, 366–370.

Christensen, R.H.B. (2018). ordinal: Regression Models for Ordinal Data.

Dodell-Feder, D., Tully, L.M., Lincoln, S.H., and Hooker, C.I. (2014). The neural basis of theory of mind and its relationship to social functioning and social anhedonia in individuals with schizophrenia. NeuroImage: Clinical *4*, 154–163.

Dudeck, M., Sosic-Vasic, Z., Otte, S., Rasche, K., Leichauer, K., Tippelt, S., Shenar, R., Klingner, S., Vasic, N., and Streb, J. (2016). The association of adverse childhood experiences and appetitive aggression with suicide attempts and violent crimes in male forensic psychiatry inpatients. Psychiatry Research *240*, 352–357.

Fett, A., Shergill, S.S., Joyce, D.W., Riedl, A., and Strobel, M. (2012). To trust or not to trust: the dynamics of social interaction in psychosis. Brain *135*, 976–984.

Foulkes, L. (2019). Sadism: Review of an elusive construct. Personality and Individual Differences *151*, 109500.

Foulkes, L., Viding, E., and McCrory, E. (2014). Social Reward Questionnaire (SRQ): development and validation. Frontiers in Psychology *5*, 289.

Freeman, D., and Fowler, D. (2009). Routes to psychotic symptoms: Trauma, anxiety and psychosis-like experiences. Psychiatry Research *169*, 107–112.

Gelman, A. (2008). Scaling regression inputs by dividing by two standard deviations. Statistics in Medicine *27*, 2865–2873.

Green, C.E.L., Freeman, D., Kuipers, E., Bebbington, P., Fowler, D., Dunn, G., and Garety, P.A. (2008). Measuring ideas of persecution and social reference: the Green et al. Paranoid Thought Scales (GPTS). Psychological Medicine *38*.

Gromann, P.M., Heslenfeld, D.J., Fett, A.-K., Joyce, D.W., Shergill, S.S., and Krabbendam, L. (2013). Trust versus paranoia: abnormal response to social reward in psychotic illness. Brain *136*, 1968–1975.

Grueber, C.E., Nakagawa, S., LAWS, R.J., and JAMIESON, I.G. (2011). Multimodel inference in ecology and evolution: challenges and solutions. J Evol Biol *24*, 699–711.

Hanewald, B., Behrens, F., Gruppe, H., Sammer, G., Gallhofer, B., Krach, S., Paulus, F.M., Rademacher, L., and Ruben Iffland, J. (2017). Anticipation of Social and Monetary Rewards in Schizophrenia. Journal of Psychiatry *20,* 1-7.

Kahneman, D., Knetsch, J.L., and Thaler, R. (1986). Fairness as a Constraint on Profit Seeking: Entitlements in the Market. The American Economic Review *76*, 728–741.

Leach, C.W., Spears, R., Branscombe, N.R., and Doosje, B. (2003). Malicious pleasure: Schadenfreude at the suffering of another group. Journal of Personality and Social Psychology *84*, 932–943.

Nimon, K., and Reio, T.G. (2011). Regression Commonality Analysis: A Technique for Quantitative Theory Building. Human Resource Development Review *10*, 329–340.

O’Meara, A., Davies, J., and Hammond, S. (2011). The psychometric properties and utility of the Short Sadistic Impulse Scale (SSIS). Psychological Assessment *23*, 523–531.

Raihani, N.J., and Bell, V. (2017a). Conflict and cooperation in paranoia: a large-scale behavioural experiment. Psychological Medicine *76*, 1–11.

Raihani, N.J., and Bell, V. (2017b). Paranoia and the social representation of others: a large-scale game theory approach. Scientific Reports *7*, 4544.

Ray Mukherjee, J., Nimon, K., Mukherjee, S., Morris, D.W., Slotow, R., and Hamer, M. (2014). Using commonality analysis in multiple regressions: a tool to decompose regression effects in the face of multicollinearity. Methods in Ecology and Evolution *5*, 320–328.

Ritsner, M.S., Ratner, Y., Mendyk, N., and Gooding, D.C. (2018). The characterization of social anhedonia and its correlates in schizophrenia and schizoaffective patients. Psychiatry Research *270*, 922–928.
